# Resistance Surveillance in *Candida albicans*: A Five-Year Antifungal Susceptibility Evaluation in a Brazilian University Hospital

**DOI:** 10.1371/journal.pone.0158126

**Published:** 2016-07-14

**Authors:** Isabela Haddad Peron, Franqueline Reichert-Lima, Ariane Fidelis Busso-Lopes, Cristiane Kibune Nagasako, Luzia Lyra, Maria Luiza Moretti, Angelica Zaninelli Schreiber

**Affiliations:** 1 Clinical Pathology Department Faculty of Medical Sciences, University of Campinas, Campinas, Sao Paulo, Brazil; 2 Internal Medicine Department, Faculty of Medical Sciences, University of Campinas, Campinas, Sao Paulo, Brazil; University of Minnesota, UNITED STATES

## Abstract

*Candida albicans* caused 44% of the overall candidemia episodes from 2006 to 2010 in our university tertiary care hospital. As different antifungal agents are used in therapy and also immunocompromised patients receive fluconazole prophylaxis in our institution, this study aimed to perform an antifungal susceptibility surveillance with the *C*.*albicans* bloodstream isolates and to characterize the fluconazole resistance in 2 non-blood *C*.*albicans* isolates by sequencing *ERG11* gene. The study included 147 *C*. *albicans* bloodstream samples and 2 fluconazole resistant isolates: one from oral cavity (LIF 12560 fluconazole MIC: 8μg/mL) and one from esophageal cavity (LIF-E10 fluconazole MIC: 64μg/mL) of two different patients previously treated with oral fluconazole. The *in vitro* antifungal susceptibility to amphotericin B (AMB), 5-flucytosine (5FC), fluconazole (FLC), itraconazole (ITC), voriconazole (VRC), caspofungin (CASP) was performed by broth microdilution methodology recommended by the Clinical and Laboratory Standards Institute documents (M27-A3 and M27-S4, CLSI). All blood isolates were classified as susceptible according to CLSI guidelines for all evaluated antifungal agents (MIC range: 0,125–1.00 μg/mL for AMB, ≤0.125–1.00 μg/mL for 5FC, ≤0.125–0.5 μg/mL for FLC, ≤0.015–0.125 μg/mL for ITC, ≤0.015–0.06 μg/mL for VRC and ≤0.015–0.125 μg/mL for CASP). In this study, we also amplified and sequenced the *ERG11* gene of LIF 12560 and LIF-E10 *C*.*albicans* isolates. Six mutations encoding distinct amino acid substitutions were found (E116D, T128K, E266D, A298V, G448V and G464S) and these mutations were previously described as associated with fluconazole resistance. Despite the large consumption of antifungals in our institution, resistant blood isolates were not found over the trial period. Further studies should be conducted, but it may be that the very prolonged direct contact with the oral antifungal agent administered to the patient from which was isolated LIF E-10, may have contributed to the development of resistance.

## Introduction

The spectrum of *Candida* infection is diverse, starting from asymptomatic colonization to oropharyngeal candidiasis (OPC), cutaneous candidiasis and invasive candidiasis–including candidemia [1]. The incidence of candidiasis has risen significantly in recent decades, and bloodstream infections due to *Candida* spp. have become an important cause of morbidity and mortality in hospitalized patients [2, 3].

Oropharyngeal and esophageal candidiasis are representative causes of morbidity in HIV-infected patients [4, 5], being the most frequent opportunistic fungal infection among these patients [6].

Strains of *C*.*albicans* that are colonizing HIV-infected patients (prior to the first episode of OPC and antifungal therapy) exhibit an increased frequency of phenotypic switching that increases the proportion of phenotypes in the colonizing population which are resistant to fluconazole [5].

The increased incidence of candidemia has been attributed to numerous factors. One important factor is an ever-expanding of immunocompromised population due to mucosal or cutaneous barrier disruption [7], defects in the number and function of neutrophils or in cell-mediated immunity [2], metabolic dysfunction, and extremes of age [7–9].

The term candidemia describes the presence of *Candida* species in the blood [10] and is the most common manifestation of invasive candidiasis [11]. Systemic candidiasis often presents initially as localized infectious symptoms and can spread to other organs through haematogenic route [12]. In all cases, the treatment with an antifungal agent is necessary [13].

Several studies [14–16] associated the high mortality rates with candidemia, demonstrating the direct proportion between high mortality and patients who were not treated with an antifungal drug. Furthermore, appropriate and timely antifungal therapy is crucial to improves treatment [7, 9, 17]. The introduction of orally active antifungal azole drugs since the 1980s, particularly fluconazole, was a significant development allowing treatment of systemic fungal infections without the problem of nephrotoxicity associated with amphotericin B treatment [18].

The current use of antifungal agents raises concerns about their potential in selecting and spreading resistant fungal strains or species [19]. Studies have reported an increasing incidence of infections caused by yeasts that either have acquired resistance or are intrinsically resistant to the drug in use [19, 20]. One of the acquired resistance mechanisms is the occurrence of alterations in the affinity of azole derivatives to cytochrome P450 14-α-demethylase (Erg11p enzyme), and has been described in different post-treatment yeast species, specially *C*.*albicans* [21]. Nucleotide mutations in *ERG11* gene result in amino acid substitutions in Erg11p [22–25] and, consequently, generate affinity alterations due to conformational changes in Erg11p, affecting the binding of azole derivatives [26].

The antifungal spectrum varies according to fungal species, such as: *C*.*albicans*, *C*.*dubliniensis* and *C*.*tropicalis* are normally susceptible to all antifungals used for the treatment of candidemia; *C*.*glabrata* is less susceptible and *C*.*krusei* is intrinsically resistant to fluconazole. Additionally, *C*.*parapsilosis* is less susceptible to the echinocandins [11, 27].

From 2006 to 2010, a retrospective study [28] was conducted in our hospital to verify the frequency and distribution of *Candida* species of blood isolates in different medical specialties. *Candida albicans* caused 44% of the overall episodes, followed by *C*.*tropicalis* (21.7%), *C*.*parapsilosis* (14.4%), *C*.*glabrata* (11.2%), and *C*.*krusei* (3.5%). (17) At this time, antifungal susceptibility was performed only for non-*C*. *albicans*. In addition, two isolates of fluconazole-resistant *C*.*albicans* were detected in non-blood clinical specimens (personal communication). As in our Hospital, different antifungal agents are use in therapy and also immunocompromised patients receive fluconazole prophylaxis, we performed an antifungal susceptibility surveillance with the *C*.*albicans* bloodstream isolates. The aims of this study were to determine the antifungal surveillance among *C*.*albicans* blood isolates, and to characterize the fluconazole resistance in non-blood *C*.*albicans* isolates by sequencing *ERG11* gene.

## Materials and Methods

The study was conducted in Hospital and Clinics of the University of Campinas, a tertiary-care university hospital that provides all major medical services as the referral hospital for five million inhabitants.

From 2006 to 2010, 147 episodes of candidemia caused by *C*. *albicans* were identified. Nosocomial candidemia was defined according to the Centers for Disease Control and Prevention (CDC) criteria [28]. Only one episode per patient was included. All blood samples were collected for routine diagnostic exams and only the *Candida albicans* isolates were evaluated in this study and no clinical information was collected. For these isolates no ethical approval was necessary. Isolates LIF 12560 and LIF E-10 are part of Cristiane Kibune Nagasako project and were obtained respectively from oral cavity and after esophageal cavity brushing. Procedure was approved by State University of Campinas School of Medical Sciences Ethics Committee under number CEP 1316/2011 –CAAE 1218.0.146.000–11.

During the period above, defined daily dose (DDD), defined as the assumed average maintenance dose per day for a drug used for its main indication in adults for the following antifungal drugs were: fluconazole: 0.2 g; voriconazole: 0.4 g; caspofungin: 50 mg, and amphotericin B: 35 mg [28, 29]. The fungal infections prophylaxis protocol established by the Hospital Infection Control Commission with fluconazole is applied to patients with acute leukemia, myelodysplasia, hematopoietic stem cell recipients, graft versus host treatment and expectation of severe and prolonged neutropenia. The route of administration may be oral or intravenous.

Two *C*.*albicans* clinical isolates from two individual patients were also included in the study: one was obtained from oral cavity (LIF 12560) and one from esophageal cavity (LIF-E10). Both patients were treated previously with oral fluconazole. This study was approved by the Ethics Committee, No.1316/2011 –CAAE 1218.0.146.000–11.

This study also investigated mutations in *ERG11* gene in the two clinical isolates of fluconazole-resistant *C*.*albicans* (LIF-E10 isolate, fluconazole MIC value: 64μg/mL; LIF 12560 isolate, fluconazole MIC value: 8μg/mL) using PCR amplification and gene sequencing.

### Yeast Identification

Blood cultures were performed by automated microbiological systems BacT/ALERT 3D FA and PF bottles (bioMérieux, Inc. Durham, NC, USA). For identification, the isolates were cultured on Sabouraud dextrose agar (SDA; Difco, Sparks, Maryland, USA) at 35°C for 48-h before the tests.

All *Candida albicans* recovered from cultures were identified according to their macromorphology on Sabouraud dextrose agar (Difco, Sparks, Maryland, USA), microscopic morphology on cornmeal Tween 80 agar, germ tube test and Vitek™ 2 (bioMérieux, France). All isolates were stored in sterile distilled water [30].

### *In Vitro* Susceptibility Testing

Antifungal susceptibility tests were performed by using the broth microdilution assay according to the methodology recommended by the Clinical and Laboratory Standards Institute (CLSI) documents M27-A3 [31] and M27-S4 [32]. *In vitro* antifungal susceptibility profiles drugs were performed using pre-prepared plates (Eiken Chemical Co., Tokyo, Japan). The MICs were obtained with the microorganism’s final concentration of 0.5x10^3^ to 2.5x10^3^ cells/mL after incubation at 35°C during 72-h. The antifungal agents analyzed were amphotericin B (AMB) (range 0.03–16 μg/mL), 5-flucytosine (5FC) (range 0.125–64 μg/mL), fluconazole (FLC) (range 0.125–64 μg/mL), itraconazole (ITC) (range 0.015–8 μg/mL), voriconazole (VRC) (range 0.015–8 μg/mL) and caspofungin (CASP), which was not available on the plate and was performed separately. CASP (Sigma-Aldrich, St. Louis, MO, USA) was dissolved in water and diluted in RPMI 1640 (Sigma-Aldrich, St. Louis, MO, USA). CASP dilutions ranged from **≤**0.015–8 μg/mL. MICs were expressed as susceptible, susceptible dose-dependent or resistant, according to the CLSI recommendations. The MIC for AMB were defined as the lowest concentration that caused 100% inhibition of growth, and the MICs other antifungal agents (5FC, FLC, VRC, ITC and CASP) were described as the lowest concentrations that produced 50% reduction in growth as compared to controls. For amphotericin B, isolates inhibited by <1 μg/mL were considered susceptible. For quality control, *Candida parapsilosis* ATCC 22019 and *Candida krusei* ATCC 6258 strains were included each time that a set of isolates was tested. The MIC_50_ and MIC_90_ were defined as MIC values that inhibited 50 and 90% of the isolates, respectively.

### *ERG11* Gene Sequencing

The *ERG11* genes from isolates were amplified by PCR using the following pairs of primers (Sigma-Aldrich, St. Louis, MO, USA): ERGSec1A (5’-TTAGTGTTTTATTGGATTCCTTGGTT-3’) with ERGSec1B (5’-TCTCATTTCATCACCAAATAAAGATC-3’), ERGSec2A (5’-ACCAGAAATTACTATTTTCACTGCTTCA-3’) with ERGSec2B (5’-AAGTCAAATCATTCAAATCACCACCT-3’), and ERGSec3A (5’-AGGTGGTGATTTGAATGATTTGACTT-3’) with ERGSec3B (5’-GAACTATAATCAGGGTCAGGCACTTT-3’). Amplification of the *ERG11* gene was performed according to Xu et al. (25) in a Veriti 96 Well Thermal Cycler (Applied Biosystems, Foster City, CA, USA) in a final volume of 25μL. Each reaction contained 50ng of DNA, 12.5μL of PCR Master Mix (Promega, Fitchburg, WI, USA) and 10mM of each primer. The reaction consisted of 5 min of initial denaturation at 94°C, 33 cycles of 1 min denaturation at 94°C, 1 min annealing at 58°C, 1 min of extension at 72°C, and a final extension of 7 min at 72°C. The PCR products were analyzed by electrophoresis in a 2% agarose gel at 100V for 30 min. The sequencing products were compared to the fluconazole-sensible standard *C*.*albicans* ATCC 90028.

## Results

### *In Vitro* Susceptibility Testing

In our study, we obtained all *C*.*albicans* blood isolates fitting on CLSI [31, 32] susceptible (S) range for all drugs, as shown in Table 1.

**Table 1:** MICs Distribution (μg/mL) of 143 *Candida albicans* bloodstream isolates against amphotericin B, 5-flucytosine, fluconazole, itraconazole, voriconazole and caspofungin.

	MICs Distribution (μg/mL)						
	0,015	0,03	0,06	0,125	0,25	0,5	1
Antifungal agent	n (%)						
Amphotericin B	-	-	-	3 (2,1)	76 (53,1)	64 (44,8)	-
5-Flucytosine	-	-	-	123 (86)	14 (9,8)	3 (2,1)	3 (2,1)
Fluconazole	-	-	-	51 (35,6)	79 (55,2)	13 (9,2)	-
Itraconazole	17 (11,9)	97 (67,8)	29 (20,3)	-	-	-	-
Voriconazole	132 (92,3)	9 (6,3)	2 (1,4)	-	-	-	-
Caspofungin	35 (24,5)	43 (30,1)	47 (32,9)	18 (12,5)	-	-	-

MICs for LIF-E10 and LIF 12560 isolates are summarized in Table 2.

**Table 2:** MIC range (μg/mL) of LIF 12560 and LIF-E10 against amphotericin B, 5-flucytosine, fluconazole, itraconazole and voriconazole.

Antifungal drugs	LIF 12560	LIF-E10	Susceptible range (S) according to CLSI (μg/mL)
**Anphotericin B**	0,25	0,25	^**__**^
**5-Flucytosine**	<0,125	<0,125	≤4
**Fluconazole**	**8 (R)**	**64 (R)**	≤2
**Itraconazole**	0,125	**0,25 (S-DD)**	≤0,125
**Voriconazole**	**0,25 (S-DD)**	**1 (R)**	≤0,125

### *ERG11* Gene Sequencing

Nucleotide sequence analysis of the *ERG11* gene of *C*.*albicans* LIF-E10 and LIF 12560 isolates revealed 6 nucleotide mutations encoding six distinct amino acid substitutions (Table 3): E116D, T128K, E266D, A298V, G448V and G464S, S1 and S2 Figs. As expected, frequent silent mutations that do not change the protein sequence were identified (data not shown).

**Table 3:** Nucleotide mutations and amino acid substitutions.

Isolates	Nucleotide position in *ERG11* gene	Nucleotide mutation	Amino acid substitution
**LIF 12560**	349	GAA→GAT (349A>T)	E116D
	384	ACA→AAA (384A>C)	T128K
	799	GAA→GAC (799A>C)	E266D
	894	GCT→GTT (894 C>T)	A298V
**LIF E10**	1343	GGG→GTG (1343G>T)	G448V
	1390	GGT→AGT (1390G>A)	G464S

## Discussion

With the aim of optimizing the treatment of *Candida* sp. infections, antifungal susceptibility testing is recognized as a very useful and important tool. In this study, we provided data on antifungal susceptibility of *C*. *albicans* blood isolates and all of the 143 isolates were susceptible to all the tested drugs. After the first isolation of *Candida* from blood culture all the subsequent isolates, were evaluated for development of resistance with fluconazole discs as a local routine surveillance protocol (data not included in this study). The longer antifungal treatment period observed between first and last *Candida* isolation for these patients was of at most 2 months. We have no information about whether these patients were classified or not in the group that received also prophylactic antifungal. In any event, this fact does not appear to be significant.

As reported in previous studies [33–36], we also observed that *C*. *albicans* causing bloodstream infections were susceptible to amphotericin B. Similarly to our data, other authors referred up to 100% of *C*.*albicans* isolated in blood cultures were susceptible to fluconazole [35–38], itraconazole [39] and voriconazole [36–38]. Caspofungin also showed potent fungicidal activity against *C*.*albicans* [37, 40, 41].

In the present study, we examined the mutations in the *ERG11* gene of two fluconazole- resistant *C*.*albicans* and we found six amino acid substitutions (E116D, T128K, E266D, A298V, G448V and G464S) that were reported previously.

The contribution of the G464S mutation in *ERG11* to fluconazole resistance, in *C*.*albicans*, has also been investigated [18, 24, 42]. The relationship of the G464S substitution in fluconazole resistance was observed by Kelly et al. (1999), studying the functional expression of *C*.*albicans* PCR-amplified *ERG11*. The G448V substitution was described by Chau et al. [43] resulting in cross-resistance to fluconazole and voriconazole. In the present study, we found that G464S substitution significantly increased fluconazole MIC, and the G448V substitution is probably associated with cross-resistance to fluconazole and voriconazole in LIF-E10 isolate.

Sanglard et al. [22] demonstrated that G464S could increase the resistance to fluconazole, itraconazole and voriconazole. Our isolate LIF-E10 showed resistance to fluconazole and voriconazole, suggesting that G464S substitution correlates with resistance to this azole drugs. The authors also described T128K, E266D and A298V [44] substitutions as associated with fluconazole resistance.

Reduced affinity between the resistant isolates (LIF-E10 and LIF 12560) and fluconazole might be occurring due to amino acid substitutions. In summary, all *ERG11* point mutations that resulted in amino acid substitutions are possibly conferring resistance to azoles, particularly fluconazole and voriconazole.

According to clinical records, LIF-E10 was isolated from an HIV-positive patient who abandoned treatment with antiretroviral and made use of fluconazole at different periods over almost seven years, prior to the isolation of the resistant microrganism. According to the literature, resistant *Candida albicans* reports from oral and esophageal mucosal from HIV-positive patients is quite common [45, 46]. Kakati et al. [45] also reported *C*. *albicans* resistant esophagitis in immunocompetent individuals.

## Conclusions

Despite the large consumption of antifungals in therapy and prophylaxis, in our institution, resistant blood isolates were not found over the trial period. However, the high incidence of candidemia due to *C*.*albicans* represents a therapeutic challenge where the continuous surveillance of the antifungal susceptibility will help to select the appropriate therapy.

The emergence of resistance of *C*. *albicans* strains to azoles may become a problem also for patients with candidiasis, as fluconazole in many countries is the most common antifungal drug prescribed for oropharyngeal and vulvovaginitis due to *Candida*.

Further studies should be conducted to prove, but it may be that the very prolonged direct contact with the oral antifungal agent administered to the patient from which was isolated LIF E-10 shown in S1 File, may have contributed to the development of resistance.

Fluconazole is a well tolerant antifungal and has high bioavailability and tissue penetration. However, prolonged periods of treatment with fluconazole can induce to mutations that express fluconazole resistance causing treatment failures.

## Supporting Information

S1 Fig *Candida albicans* LIF 12560 nucleotide mutations and amino acid substitutions. **A**: A298V amino acid substitution. **B**: E116D amino acid substitution. **C**: E266D amino acid substitution. **D**: T128K amino acid substitution.
(TIF)

S2 Fig *Candida albicans* LIF E-10 nucleotide mutations and amino acid substitutions. **A**: G448V amino acid substitution. **B**: G464S amino acid substitution.
(TIF)

S1 File Isolates LIF E-10 (A) and LIF 12560 (B) patients data provided to be released.
(DOCX)

S1 Table Antifungal susceptibility tests (MIC results). (DOC)
