# Supplementary figures and images for: Resistance Surveillance in Candida albicans: A Five-Year Antifungal Susceptibility Evaluation in a Brazilian University Hospital
Source: PLoS One. 2016 Jul 14;11(7):e0158126. doi: 10.1371/journal.pone.0158126 (PMC4945058; doi:10.1371/journal.pone.0158126)

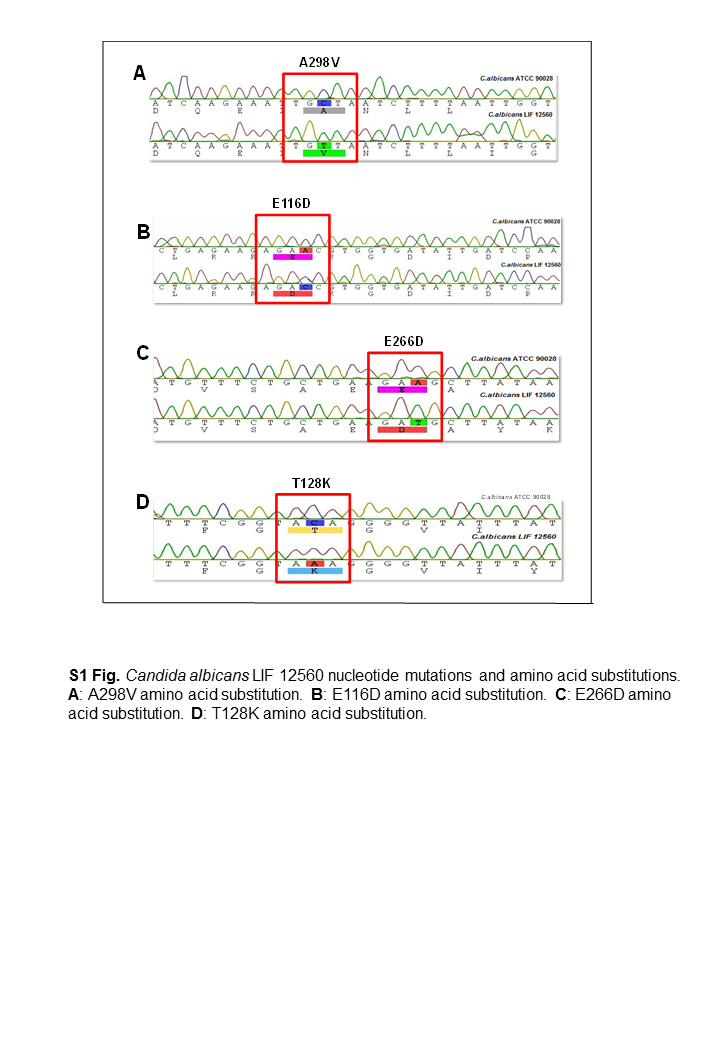

Supplement: S1 Fig — A: A298V amino acid substitution. B: E116D amino acid substitution. C: E266D amino acid substitution. D: T128K amino acid substitution. (TIF) [file pone.0158126.s001.tif]

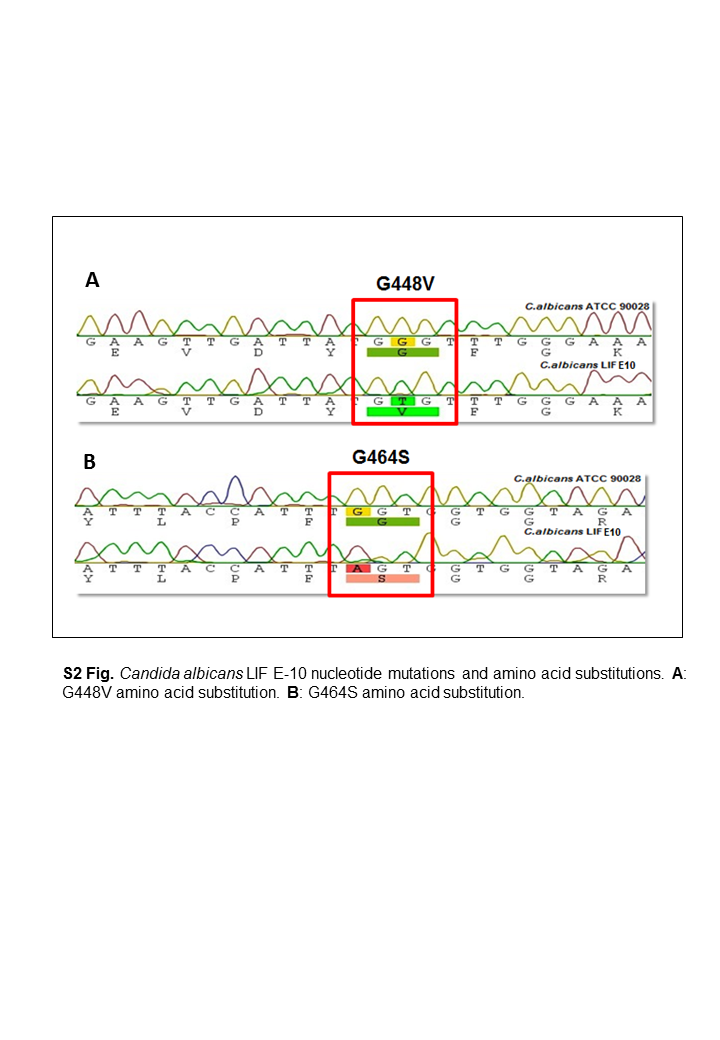

Supplement: S2 Fig — A: G448V amino acid substitution. B: G464S amino acid substitution. (TIF) [file pone.0158126.s002.tif]
