# Supplementary material for: Resistance Surveillance in Candida albicans: A Five-Year Antifungal Susceptibility Evaluation in a Brazilian University Hospital
Source: PLoS One. 2016 Jul 14;11(7):e0158126. doi: 10.1371/journal.pone.0158126 (PMC4945058; doi:10.1371/journal.pone.0158126)
Supplement: S1 File — Isolates LIF E-10 (A) and LIF 12560 (B) patients data provided to be released. (DOCX) [file pone.0158126.s003.docx]

**S1 File. Isolates LIF E-10 (A) and LIF 12560 (B) patients data provided to be released**

1. **LIF E-10:** isolated from female patient, 22 years, HIV without HAART (treatment dropout), with dysphagia / odynophagia and oral candidiasis CD4 148 CV 17.000 and received oral fluconazole before LIF E 10 recovery.

History dates raised:

11/21/2005 Fluconazole 150mg/day/7days because of oral candidiasis and with dysphagia

01/31/2008 Fluconazole 150mg/week/6 month because of onychomycosis

03/10/2009 Fluconazole 150mg/day/14 days because of oral candidiasis and dysphagia

08/03/2009 Fluconazole 200mg/day/14 days because of oral candidiasis

05/29/2012 LIF E 10 isolate recovery from esophageal cavity brushing

1. **LIF 12560**

Isolated from HIV-positive patient oral cavity. Patient presenting oral candidiasis. Oral communication that the patient received fluconazole previously to microorganism recovery. Other data were not available.

The above data were collected on patients and used for discussion of this study. It is possible that these patients continue to be followed up in our hospital. For further information please e-mail correspondent author (zaninele@fcm.unicamp.br).
