# Supplementary material for: Resistance Surveillance in Candida albicans: A Five-Year Antifungal Susceptibility Evaluation in a Brazilian University Hospital
Source: PLoS One. 2016 Jul 14;11(7):e0158126. doi: 10.1371/journal.pone.0158126 (PMC4945058; doi:10.1371/journal.pone.0158126)
Supplement: S1 Table — (DOC) [file pone.0158126.s004.doc]

|  |  | **Dry Plate System** | | | | | | **Manual dilution** |
| --- | --- | --- | --- | --- | --- | --- | --- | --- |
| **Number** | **Year** | **MCFG IC50** | **AMPH IC100** | **5-FC IC50** | **FLCZ IC50** | **ITCZ IC50** | **VRCZ IC50** | **CASP IC50** |
| 7585 | 2006 | **<0,015** | **0,5** | **0,25** | **≤0,125** | **0,015** | **<0,015** | **0,03** |
| 7635 | 2006 | **<0,015** | **0,5** | **0,125** | **0,25** | **0,03** | **<0,015** | **≤0,015** |
| 7653 | 2006 | **<0,015** | **0,5** | **<0,125** | **0,25** | **0,03** | **≤0,015** | **≤0,015** |
| 7662 | 2006 | **<0,015** | **0,5** | **<0,125** | **0,25** | **0,03** | **≤0,015** | **0,06** |
| 7664 | 2006 | **<0,015** | **0,25** | **<0,125** | **0,25** | **0,03** | **<0,015** | **0,06** |
| 7676 | 2006 | **<0,015** | **0,25** | **<0,125** | **0,25** | **0,03** | **<0,015** | **≤0,015** |
| 7680 | 2006 | **≤0,015** | **0,5** | **0,125** | **≤0,125** | **0,03** | **<0,015** | **0,03** |
| 7707 | 2006 | **≤0,015** | **0,5** | **≤0,125** | **0,25** | **0,03** | **≤0,015** | **0,03** |
| 7709 | 2006 | **<0,015** | **0,25** | **<0,125** | **0,25** | **0,03** | **≤0,015** | **≤0,015** |
| 7794 | 2006 | **<0,015** | **0,5** | **≤0,125** | **0,25** | **0,03** | **<0,015** | **≤0,015** |
| 7835 | 2006 | **<0,015** | **0,5** | **<0,125** | **≤0,125** | **0,03** | **≤0,015** | **≤0,015** |
| 7866 | 2006 | **<0,015** | **0,5** | **0,25** | **≤0,125** | **0,03** | **≤0,015** | **0,06** |
| 7874 | 2006 | **<0,015** | **0,25** | **<0,125** | **≤0,125** | **0,015** | **≤0,015** | **0,03** |
| 7882 | 2006 | **<0,015** | **0,5** | **<0,125** | **0,25** | **0,03** | **≤0,015** | **≤0,015** |
| 8072 | 2006 | **<0,015** | **0,25** | **<0,125** | **0,25** | **0,06** | **≤0,015** | **0,03** |
| 8123 | 2006 | **≤0,015** | **0,25** | **<0,125** | **0,25** | **0,03** | **≤0,015** | **≤0,015** |
| 8144 | 2006 | **<0,015** | **0,25** | **<0,125** | **≤0,125** | **0,03** | **≤0,015** | **≤0,015** |
| 8210 | 2006 | **<0,015** | **0,5** | **<0,125** | **≤0,125** | **0,03** | **≤0,015** | **≤0,015** |
| 8228 | 2006 | **≤0,015** | **0,5** | **<0,125** | **0,25** | **0,03** | **≤0,015** | **≤0,015** |
| 8244 | 2006 | **<0,015** | **0,5** | **≤0,125** | **0,25** | **0,06** | **≤0,015** | **0,03** |
| 8381 | 2006 | **<0,015** | **0,25** | **0,25** | **0,5** | **0,03** | **<0,015** | **0,03** |
| 8399 | 2006 | **<0,015** | **0,5** | **0,125** | **0,25** | **0,06** | **≤0,015** | **0,03** |
| 8407 | 2006 | **<0,015** | **0,5** | **<0,125** | **0,25** | **0,03** | **≤0,015** | **≤0,015** |
| 8432 | 2006 | **<0,015** | **0,5** | **<0,125** | **0,25** | **0,03** | **≤0,015** | **≤0,015** |
| 8438 | 2006 | **<0,015** | **0,5** | **<0,125** | **0,25** | **0,03** | **≤0,015** | **0,03** |
| 8462 | 2006 | **<0,015** | **0,25** | **<0,125** | **0,125** | **0,015** | **<0,015** | **≤0,015** |
| 8548 | 2007 | **<0,015** | **0,25** | **≤0,125** | **0,125** | **0,03** | **0,015** | **≤0,015** |
| 8550 | 2007 | **<0,015** | **0,25** | **<0,125** | **0,5** | **0,03** | **0,015** | **≤0,015** |
| 8671 | 2007 | **<0,015** | **0,25** | **<0,125** | **0,125** | **0,015** | **<0,015** | **≤0,015** |
| 8704 | 2007 | **<0,015** | **0,5** | **≤0,125** | **0,25** | **0,06** | **<0,015** | **≤0,015** |
| 8733 | 2007 | **<0,015** | **0,5** | **<0,125** | **≤0,125** | **≤0,015** | **<0,015** | **0,03** |
| 8737 | 2007 | **≤0,015** | **0,25** | **<0,125** | **≤0,125** | **0,03** | **≤0,015** | **0,03** |
| 8777 | 2007 | **<0,015** | **0,25** | **≤0,125** | **≤0,125** | **0,03** | **<0,015** | **≤0,015** |
| 8780 | 2007 | **<0,015** | **0,25** | **0,25** | **0,25** | **0,03** | **<0,015** | **≤0,015** |
| 8982 | 2007 | **<0,015** | **0,5** | **<0,125** | **0,25** | **0,03** | **<0,015** | **0,06** |
| 8991 | 2007 | **<0,015** | **0,25** | **<0,125** | **0,25** | **0,06** | **≤0,015** | **≤0,015** |
| 9028 | 2007 | **<0,015** | **0,5** | **≤0,125** | **≤0,125** | **0,03** | **≤0,015** | **≤0,015** |
| 9044 | 2007 | **0,03** | **0,25** | **<0,125** | **0,25** | **0,03** | **<0,015** | **0,03** |
| 9050 | 2007 | **≤0,015** | **0,5** | **<0,125** | **≤0,125** | **0,03** | **≤0,015** | **≤0,015** |
| 9059 | 2007 | **<0,015** | **0,5** | **1** | **≤0,125** | **0,03** | **≤0,015** | **0,03** |
| 9197 | 2007 | **<0,015** | **0,25** | **≤0,125** | **0,25** | **0,03** | **≤0,015** | **≤0,015** |
| 9211 | 2007 | **≤0,015** | **0,5** | **<0,125** | **0,25** | **0,03** | **≤0,015** | **≤0,015** |
| 9213 | 2007 | **<0,015** | **0,5** | **<0,125** | **≤0,125** | **0,03** | **<0,015** | **0,06** |
| 9215 | 2007 | **<0,015** | **0,5** | **≤0,125** | **≤0,125** | **0,03** | **≤0,015** | **0,03** |
| 9309 | 2007 | **<0,015** | **0,5** | **0,25** | **0,25** | **0,015** | **<0,015** | **0,03** |
| 9317 | 2007 | **≤0,015** | **0,25** | **<0,125** | **≤0,125** | **0,03** | **≤0,015** | **0,03** |
| 9334 | 2007 | **<0,015** | **0,5** | **<0,125** | **≤0,125** | **0,03** | **<0,015** | **0,03** |
| 9418 | 2008 | **≤0,015** | **0,25** | **≤0,125** | **0,25** | **0,03** | **≤0,015** | **0,03** |
| 9484 | 2008 | **<0,015** | **0,5** | **<0,125** | **0,25** | **0,06** | **0,03** | **0,03** |
| 9494 | 2008 | **<0,015** | **0,125** | **<0,125** | **≤0,125** | **0,03** | **<0,015** | **0,03** |
| 9515 | 2008 | **<0,015** | **0,25** | **<0,125** | **<0,125** | **≤0,015** | **<0,015** | **0,03** |
| 9560 | 2008 | **<0,015** | **0,125** | **<0,125** | **0,25** | **0,03** | **≤0,015** | **0,06** |
| 9564 | 2008 | **<0,015** | **0,25** | **<0,125** | **0,25** | **0,015** | **<0,015** | **0,03** |
| 9566 | 2008 | **<0,015** | **0,25** | **<0,125** | **0,25** | **0,03** | **≤0,015** | **≤0,015** |
| 9580 | 2008 | **<0,015** | **0,25** | **<0,125** | **0,125** | **0,015** | **<0,015** | **0,03** |
| 9717 | 2008 | **<0,015** | **0,5** | **<0,125** | **0,25** | **0,03** | **≤0,015** | **0,03** |
| 9731 | 2008 | **≤0,015** | **0,25** | **<0,125** | **0,25** | **0,03** | **≤0,015** | **0,03** |
| 9734 | 2008 | **<0,015** | **0,25** | **0,25** | **≤0,125** | **0,06** | **≤0,015** | **0,03** |
| 9735 | 2008 | **<0,015** | **0,25** | **0,25** | **0,5** | **0,03** | **≤0,015** | **0,03** |
| 9760 | 2008 | **<0,015** | **0,25** | **1** | **0,25** | **0,03** | **≤0,015** | **0,06** |
| 9770 | 2008 | **<0,015** | **0,5** | **<0,125** | **0,5** | **0,06** | **≤0,015** | **0,125** |
| 9777 | 2008 | **<0,015** | **0,25** | **<0,125** | **≤0,125** | **0,03** | **≤0,015** | **0,125** |
| 9789 | 2008 | **<0,015** | **0,5** | **<0,125** | **≤0,125** | **0,03** | **≤0,015** | **0,06** |
| 9831 | 2008 | **<0,015** | **0,5** | **<0,125** | **0,25** | **0,03** | **≤0,015** | **0,03** |
| 9840 | 2008 | **<0,015** | **0,5** | **<0,125** | **≤0,125** | **0,03** | **≤0,015** | **0,06** |
| 9846 | 2008 | **<0,015** | **0,5** | **<0,125** | **≤0,125** | **0,03** | **≤0,015** | **0,03** |
| 9891 | 2008 | **<0,015** | **0,5** | **<0,125** | **0,25** | **0,03** | **≤0,015** | **0,03** |
| 9923 | 2008 | **<0,015** | **0,25** | **<0,125** | **≤0,125** | **0,03** | **≤0,015** | **0,03** |
| 9940 | 2008 | **<0,015** | **0,5** | **<0,125** | **≤0,125** | **0,06** | **≤0,015** | **0,06** |
| 9968 | 2008 | **<0,015** | **0,25** | **0,25** | **0,25** | **0,03** | **≤0,015** | **≤0,015** |
| 9984 | 2008 | **<0,015** | **0,25** | **0,25** | **≤0,125** | **<0,015** | **<0,015** | **0,03** |
| 9994 | 2008 | **≤0,015** | **0,5** | **0,25** | **0,25** | **0,03** | **≤0,015** | **0,03** |
| 9996 | 2008 | **<0,015** | **0,5** | **0,5** | **0,25** | **0,03** | **≤0,015** | **0,03** |
| 10007 | 2008 | **<0,015** | **0,5** | **<0,125** | **≤0,125** | **0,03** | **≤0,015** | **0,125** |
| 10012 | 2008 | **<0,015** | **0,5** | **≤0,125** | **0,25** | **0,06** | **≤0,015** | **0,125** |
| 10014 | 2008 | **<0,015** | **0,25** | **≤0,125** | **0,25** | **0,03** | **≤0,015** | **0,06** |
| 10020 | 2008 | **<0,015** | **0,125** | **<0,125** | **0,25** | **0,03** | **≤0,015** | **N.R.** |
| 10027 | 2008 | **<0,015** | **0,25** | **≤0,125** | **0,25** | **0,03** | **≤0,015** | **0,06** |
| 10031 | 2008 | **<0,015** | **0,25** | **<0,125** | **≤0,125** | **0,03** | **≤0,015** | **0,06** |
| 10032 | 2008 | **<0,015** | **0,5** | **≤0,125** | **≤0,125** | **0,03** | **≤0,015** | **0,06** |
| 10042 | 2008 | **<0,015** | **0,25** | **<0,125** | **≤0,125** | **0,03** | **≤0,015** | **0,06** |
| 10043 | 2008 | **<0,015** | **0,25** | **<0,125** | **0,25** | **0,06** | **≤0,015** | **0,06** |
| 10058 | 2008 | **<0,015** | **0,25** | **<0,125** | **0,25** | **0,015** | **<0,015** | **0,06** |
| 10159 | 2008 | **<0,015** | **0,25** | **<0,125** | **0,25** | **0,03** | **≤0,015** | **0,125** |
| 10440 | 2009 | **<0,015** | **0,25** | **<0,125** | **≤0,125** | **0,03** | **≤0,015** | **0,06** |
| 10449 | 2009 | **<0,015** | **0,5** | **<0,125** | **0,25** | **0,03** | **≤0,015** | **0,06** |
| 10450 | 2009 | **<0,015** | **0,5** | **<0,125** | **0,25** | **0,06** | **≤0,015** | **0,06** |
| 10459 | 2009 | **<0,015** | **0,5** | **<0,125** | **≤0,125** | **0,03** | **≤0,015** | **0,03** |
| 10480 | 2009 | **<0,015** | **0,5** | **<0,125** | **0,25** | **0,06** | **≤0,015** | **0,125** |
| 10496 | 2009 | **<0,015** | **0,5** | **<0,125** | **0,25** | **0,03** | **≤0,015** | **0,03** |
| 10531 | 2009 | **<0,015** | **0,5** | **<0,125** | **≤0,125** | **0,03** | **≤0,015** | **0,06** |
| 10534 | 2009 | **<0,015** | **0,5** | **<0,125** | **0,25** | **0,06** | **≤0,015** | **0,06** |
| 10588 | 2009 | **<0,015** | **0,25** | **<0,125** | **≤0,125** | **≤0,015** | **≤0,015** | **0,03** |
| 10593 | 2009 | **<0,015** | **0,25** | **<0,125** | **0,125** | **<0,015** | **<0,015** | **0,06** |
| 10594 | 2009 | **<0,015** | **0,25** | **≤0,125** | **0,25** | **0,03** | **≤0,015** | **0,06** |
| 10599 | 2009 | **<0,015** | **0,5** | **0,5** | **≤0,125** | **0,03** | **<0,015** | **0,03** |
| 10604 | 2009 | **<0,015** | **0,25** | **<0,125** | **0,25** | **0,03** | **≤0,015** | **0,06** |
| 10681 | 2009 | **<0,015** | **0,25** | **<0,125** | **0,25** | **0,03** | **0,03** | **0,06** |
| 10691 | 2009 | **<0,015** | **0,25** | **≤0,125** | **0,25** | **0,03** | **<0,015** | **0,06** |
| 10696 | 2009 | **<0,015** | **0,5** | **<0,125** | **≤0,125** | **0,03** | **≤0,015** | **0,03** |
| 10721 | 2009 | **<0,015** | **0,5** | **≤0,125** | **≤0,125** | **0,03** | **≤0,015** | **0,06** |
| 10737 | 2009 | **<0,015** | **0,25** | **<0,125** | **0,25** | **≤0,015** | **≤0,015** | **0,06** |
| 10739 | 2009 | **<0,015** | **0,5** | **<0,125** | **≤0,125** | **0,03** | **≤0,015** | **0,06** |
| 10759 | 2009 | **<0,015** | **0,5** | **<0,125** | **0,5** | **0,06** | **0,03** | **0,125** |
| 10771 | 2009 | **<0,015** | **0,5** | **<0,125** | **0,25** | **0,03** | **≤0,015** | **0,125** |
| 10776 | 2009 | **<0,015** | **0,5** | **≤0,125** | **0,25** | **0,015** | **<0,015** | **0,06** |
| 10798 | 2009 | **<0,015** | **0,5** | **0,25** | **0,25** | **0,03** | **<0,015** | **0,06** |
| 10803 | 2009 | **<0,015** | **0,25** | **<0,125** | **0,25** | **0,03** | **<0,015** | **0,06** |
| 10807 | 2009 | **<0,015** | **0,25** | **0,25** | **0,125** | **0,03** | **<0,015** | **0,06** |
| 10851 | 2009 | **<0,015** | **0,25** | **<0,125** | **0,25** | **0,03** | **≤0,015** | **0,06** |
| 10875 | 2009 | **<0,015** | **0,25** | **<0,125** | **0,25** | **0,06** | **≤0,015** | **0,03** |
| 10913 | 2009 | **≤0,015** | **0,25** | **<0,125** | **≤0,125** | **≤0,015** | **≤0,015** | **0,06** |
| 10918 | 2009 | **<0,015** | **0,5** | **≤0,125** | **≤0,125** | **≤0,015** | **<0,015** | **≤0,015** |
| 10927 | 2009 | **<0,015** | **0,25** | **<0,125** | **0,5** | **0,06** | **≤0,015** | **0,125** |
| 10950 | 2009 | **<0,015** | **0,25** | **≤0,125** | **0,25** | **0,03** | **≤0,015** | **≤0,015** |
| 10951 | 2009 | **<0,015** | **0,5** | **<0,125** | **0,5** | **0,06** | **0,03** | **0,125** |
| 10985 | 2009 | **<0,015** | **0,25** | **<0,125** | **0,25** | **0,03** | **≤0,015** | **≤0,015** |
| 10988 | 2009 | **≤0,015** | **0,25** | **<0,125** | **0,5** | **0,06** | **0,06** | **0,06** |
| 11018 | 2009 | **<0,015** | **0,25** | **0,125** | **0,25** | **0,06** | **0,03** | **0,06** |
| 11027 | 2009 | **<0,015** | **0,5** | **0,125** | **0,25** | **0,03** | **≤0,015** | **≤0,015** |
| 11054 | 2010 | **≤0,015** | **0,25** | **<0,125** | **≤0,125** | **0,03** | **≤0,015** | **≤0,015** |
| 11060 | 2010 | **<0,015** | **0,25** | **<0,125** | **0,5** | **0,06** | **≤0,015** | **0,06** |
| 11067 | 2010 | **<0,015** | **0,25** | **0,25** | **0,5** | **0,03** | **≤0,015** | **0,03** |
| 11072 | 2010 | **≤0,015** | **0,5** | **<0,125** | **0,25** | **0,06** | **0,03** | **0,03** |
| 11080 | 2010 | **<0,015** | **0,5** | **<0,125** | **≤0,125** | **0,03** | **≤0,015** | **0,06** |
| 11081 | 2010 | **<0,015** | **0,25** | **0,25** | **0,5** | **0,06** | **0,03** | **≤0,015** |
| 11087 | 2010 | **<0,015** | **0,25** | **<0,125** | **0,25** | **0,03** | **≤0,015** | **0,06** |
| 11088 | 2010 | **≤0,015** | **0,25** | **≤0,125** | **≤0,125** | **0,03** | **≤0,015** | **≤0,015** |
| 11093 | 2010 | **≤0,015** | **0,25** | **≤0,125** | **0,25** | **0,03** | **≤0,015** | **0,03** |
| 11106 | 2010 | **<0,015** | **0,25** | **<0,125** | **≤0,125** | **0,03** | **≤0,015** | **0,06** |
| 11109 | 2010 | **<0,015** | **0,25** | **0,5** | **0,25** | **0,03** | **≤0,015** | **0,125** |
| 11112 | 2010 | **<0,015** | **0,25** | **<0,125** | **0,25** | **0,03** | **0,03** | **0,125** |
| 11125 | 2010 | **<0,015** | **0,25** | **<0,125** | **0,25** | **0,03** | **≤0,015** | **0,125** |
| 11133 | 2010 | **<0,015** | **0,5** | **<0,125** | **0,25** | **0,03** | **≤0,015** | **0,125** |
| 11144 | 2010 | **<0,015** | **0,25** | **<0,125** | **0,5** | **0,06** | **≤0,015** | **0,125** |
| 11153 | 2010 | **≤0,015** | **0,25** | **<0,125** | **0,25** | **0,06** | **≤0,015** | **0,125** |
| 11192 | 2010 | **≤0,015** | **0,5** | **<0,125** | **0,25** | **0,03** | **≤0,015** | **0,06** |
| 11197 | 2010 | **<0,015** | **0,25** | **<0,125** | **0,5** | **0,06** | **0,06** | **0,06** |
| 11199 | 2010 | **<0,015** | **0,25** | **<0,125** | **0,25** | **0,06** | **≤0,015** | **0,06** |
| 11202 | 2010 | **<0,015** | **0,25** | **1** | **0,25** | **0,06** | **≤0,015** | **0,125** |
| 11208 | 2010 | **<0,015** | **0,25** | **<0,125** | **0,25** | **0,06** | **≤0,015** | **0,125** |
| 11215 | 2010 | **<0,015** | **0,25** | **<0,125** | **0,25** | **0,03** | **≤0,015** | **0,06** |
| 11245 | 2010 | **<0,015** | **0,25** | **<0,125** | **0,25** | **0,03** | **0,03** | **0,06** |
